# Application of Zero-Dimensional Nanomaterials in Biosensing

**DOI:** 10.3389/fchem.2020.00320

**Published:** 2020-04-17

**Authors:** Zhengdi Wang, Tingting Hu, Ruizheng Liang, Min Wei

**Affiliations:** State Key Laboratory of Chemical Resource Engineering, Beijing Advanced Innovation Center for Soft Matter Science and Engineering, Beijing University of Chemical Technology, Beijing, China

**Keywords:** 0D nanomaterials, biosensing, ion detection, disease diagnosis, pathogen detection

## Abstract

Zero-dimensional (0D) nanomaterials, including graphene quantum dots (GQDs), carbon quantum dots (CQDs), fullerenes, inorganic quantum dots (QDs), magnetic nanoparticles (MNPs), noble metal nanoparticles, upconversion nanoparticles (UCNPs) and polymer dots (Pdots), have attracted extensive research interest in the field of biosensing in recent years. Benefiting from the ultra-small size, quantum confinement effect, excellent physical and chemical properties and good biocompatibility, 0D nanomaterials have shown great potential in ion detection, biomolecular recognition, disease diagnosis and pathogen detection. Here we first introduce the structures and properties of different 0D nanomaterials. On this basis, recent progress and application examples of 0D nanomaterials in the field of biosensing are discussed. In the last part, we summarize the research status of 0D nanomaterials in the field of biosensing and anticipate the development prospects and future challenges in this field.

## Introduction

Nanotechnology has been a fast-growing field in the past few decades. By changing the chemical composition, atomic arrangement, or dimension of nanomaterials, many nanomaterials with alterations in physical and physicochemical properties are produced (Carneiro et al., 2019; Raja et al., 2019; Vikrant et al., 2019). Since the term “nano” was coined by Norio Taniguchi in 1974, zero-dimensional (0D) nanomaterials have been hailed as the forerunner of nanotechnology. Due to the inherent structural properties of 0D nanomaterials, such as ultra-small sizes and high surface-to-volume ratios, they have more active edge sites per unit mass. The edge and quantum confinement effects of 0D nanomaterials endow them with more improved or novel properties such as high photoluminescence (PL) quantum efficiency and chemiluminescence (Jiang and Tian, 2018; Chen J. B. et al., 2019; Farzin et al., 2019; Pirzada and Altintas, 2019). Up to now, various 0D nanomaterials have been extensively explored. For instance, graphene quantum dots (GQDs) (Qian et al., 2014; Lu et al., 2018; Yan et al., 2019), carbon quantum dots (CQDs) (Zheng et al., 2015; Li et al., 2018b; Pandit et al., 2019), fullerenes (Barberis et al., 2015; Zhang C. et al., 2018), inorganic quantum dots (QDs) (Freeman et al., 2012; Li et al., 2018a; Robidillo et al., 2019), magnetic nanoparticles (MNPs) (Yu et al., 2017; Bragina et al., 2019), noble metal nanoparticles (Yang C. T. et al., 2016; Jain and Chauhan, 2017; Bagheri et al., 2018), upconversion nanoparticles (UCNPs) (Cheng Z. H. et al., 2019; Gu et al., 2019) and polymer dots (Pdots) (Ou et al., 2019; Zhang H. et al., 2019) are all representative 0D nanomaterials with potential applications in materials science (Zhang X. et al., 2014), photovoltaic science (Guo et al., 2010), catalysis (Xu et al., 2015), energy (Guldi and Sgobba, 2011), sensing (Ramanathan et al., 2019), biomedicine (Yao et al., 2018) and nanodevices (Zhang Y. et al., 2014).

By changing the size of nanomaterials and converting them to zero-dimensional structures, the newly fabricated structure can be given novel properties that differ from those of higher-dimensional materials (Liang et al., 2014a). Compared with bulk high-dimensional nanomaterials, 0D nanomaterials are mostly spherical or quasi-spherical nanoparticles with a diameter of less than 100 nm (Liu J. N. et al., 2017; Chen J. B. et al., 2019; Sondhi et al., 2020). With such novel properties as optical stability, wavelength-dependent photoluminescence, chemical inertness, cellular permeability and biocompatibility, 0D nanomaterials offer great adaptability to biomedical applications such as nanomedicine, cosmetics, bioelectronics, biosensor and biochip (Koh and Josephson, 2009; Yao et al., 2018). Biosensor is considered as a reliable and usually portable tool for the rapid and cost-effective determination of analytes including biomolecules, antigens, proteins, biotoxins, DNA, viruses, bacteria and others. One of the main challenges in the field of biosensor is to develop highly sensitive biosensors by increasing the active surface area, electrochemical activity and conductivity or optical performance of biosensors (Pirzada and Altintas, 2019; Dhenadhayalan et al., 2020). Therefore, 0D nanomaterials as a powerful sensing material are utilized as an important probe to increase the sensitivity of biosensors and thus enhance their analytical performance due to their conductive properties and special optical properties (Carneiro et al., 2019; Sondhi et al., 2020).

Over the past few years, an increasing number of studies on 0D nanomaterials regarding their application in biosensors have been reported (Jiang and Tian, 2018; Chen J. B. et al., 2019), but few have provided a comprehensive overview of this application, which possesses great significance to the biosensing development of 0D nanomaterials. Therefore, this review intends to present an overview of the most frequently used 0D nanomaterials in the development of biosensing. Firstly, the structures and properties of these 0D nanomaterials are introduced. Subsequently, their application in ion, biomolecule and pathogen detection and disease diagnosis are discussed in detail for a comprehensive understanding of biosensors based on 0D nanomaterials. In the end, we summarize the research status of 0D nanomaterials in the field of biosensing, and anticipate the development prospects and future challenges in this field.

## 0D Carbon-Based Nanomaterials

Carbon-based nanomaterials are one of the most widely studied materials in the field of nanotechnology due to their low cost of mass production, low intrinsic toxicity and multifunctional surface functionalization (Panwar et al., 2019). Because of these excellent properties, carbon-based nanomaterials have become promising alternatives to other nanomaterials in a variety of biological applications, such as imaging, sensing and drug delivery (Xu Q. et al., 2019). Among all these carbon-based nanomaterials, 0D carbon-based nanomaterials have inimitable electrical, optical properties, low toxicity and high quantum yield; therefore can be used to produce micro-sensors with superior performance and low power consumption (Shi et al., 2019; Zhou et al., 2019). In this section, we will give a detailed introduction to the latest progress in the application of 0D carbon-based nanomaterials in the field of biosensing.

### Graphene Quantum Dots

Graphene quantum dots (GQDs) are a new type of 0D graphene nanomaterials characterized by atom-thin graphitized planes (usually 1 or 2 layers, not exceeding 2 nanometers in thickness) and small transverse dimensions (<10 nanometers in general) (Chung et al., 2019; Li et al., 2019). One of the outstanding features of GQDs is PL. A large number of studies have demonstrated that the PL excitation and emission wavelength of GQDs can be changed by adjusting their dimension, morphology or dopant. The tunable PL property enables GQDs' use in bioimaging and biosensing. In addition, there are plenty of oxygen-rich functional groups at the edge of GQDs, which contributes to their good water solubility and biocompatibility (Sun et al., 2013). Furthermore, due to such advantages as anti-bleaching, luminescence stability and good conductivity, the application of GQDs in the field of biosensing has been further expanded (Zhu et al., 2015). Now, GQDs-based sensors are widely used in the detection of various ions and biomarkers as well as the diagnosis of major diseases.

By virtue of their PL properties, GQDs have been used in the manufacture of many optical biosensors to detect various metal ions (Chung et al., 2019). For example, Pathan et al. constructed an aggregation-induced enhanced PL sensing system based on magnetic graphene oxide quantum dots (Fe-GQDs) to sensitively and selectively detect arsenic ions in contaminated water. The detection limit of Fe-GQDs for As^3+^ was 5.1 ppb, which is lower than the WHO allowable limit for arsenic in drinking water (10 μg/L) (Pathan et al., 2019). In another work, Qian et al. synthesized a fluorescent sensor based on GQDs–aptamer probe and graphene oxide (GO) to detect Pb^2+^. The GQDs–aptamer served as the fluorophore for the detection of Pb^2+^, and the unique electron transfer between GQDs and GO was used to achieve the efficient detection of Pb^2+^. This nanosensor possessed a linear range of 400.0 nM and a detection limit as low as 0.6 nM. Due to the excellent biocompatibility of GQDs and GO, this sensing system was expected to be used for the detection of Pb^2+^ *in vivo* and *in vitro* (Qian et al., 2015).

In addition, GQDs-based biosensors play a significant role in the detection of biomarkers, such as ascorbic acid (AA), dopamine, DNA and amino acid. In a recent study, a sensitive and rapid fluorescence turn-on nanosensor based on orange emission GQDs was developed for the detection of AA. AA could consume hydroxyl radicals and recover the fluorescence of GQDs quenched by o-benzoquinone. Such a fluorescence switch mode provided the sensor with such advantages as universality and high selectivity. Besides, no heavy metal element was added to the system and thus avoided heavy metal contamination. According to the experimental results, the detection limit of this GQDs-based biosensor on AA was 0.32 μM, which was lower than that of other fluorescence biosensors. Glutathione (GSH) monitoring has received considerable attention for its vital role in human diseases (Liu H. et al., 2017). Yan et al. designed a fluorescence “turn–off–on” biosensor based on GQDs–MnO_2_ nanosheets for the ultrasensitive detection of GSH in living cells. The fluorescence intensity of GQDs was quenched by the fluorescence resonance energy transfer between MnO_2_ and GQDs. After the nanometer sensor entered the cell, GSH could reduce MnO_2_ nanosheets to Mn^2+^ cation so as to release GQDs and sufficiently recover the fluorescence signal. This sensing platform displayed a sensitive response to GSH with an ultralow detection limit of 150 nM (Yan et al., 2016).

**Figure 1:**
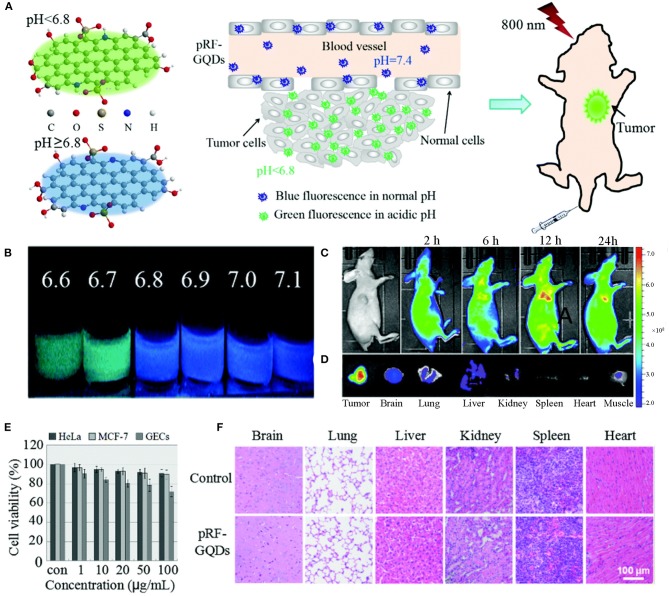
**(A)** Schematic diagrams of pRF-GQDs at different pH values and their application in tumor imaging. **(B)** Digital images of pRF-GQDs at different pH values. **(C)** Fluorescence images of a HeLa tumor-bearing mouse after intravenous injection of pRF-GQDs. **(D)** *Ex vivo* imaging of major organs from a mouse treated with pRF-GQDs. **(E)** Cytotoxicity of pRF-GQDs on indicated cells. **(F)** H&E stained indicated tissue slices from two groups of healthy mice after 15 d post-treatment. Reproduced with permission from Fan et al. (2017). Copyright 2017, Royal Society of Chemistry.

Apart from the detection of small molecules, GQDs-based biosensors can also be used as tools to diagnose cancer (Xi et al., 2016). Because tumors can produce lactic acid and conduct adenosine triphosphate hydrolysis under anaerobic and energy-deficient conditions, their pH values are lower than those of healthy tissues. This characteristic has been clinically exploited for efficient cancer diagnosis. A pH-responsive fluorescent sulfur-nitrogen-doped GQDs probe (pRF-GQDs) was constructed to distinguish tumors from normal tissues (Figure 1). The pRF-GQDs showed green PL in pH below 6.8 and transited into blue PL in pH overtop 6.8, a value matching the acidic extracellular microenvironment in solid tumors. The fluorescence switch was reversible and the fluorescence intensity was related to the degree of acidosis. The prepared pRF-GQDs showed excellent stability. The fluorescence intensity remained unchanged after continuous irradiation for 24 h. After the injection of pRF-GQDs, the tumor sites of tumor-bearing mice showed a strong green PL signal (Fan et al., 2017). This GQDs-based biosensor has great potential to be used as a universal fluorescent probe to tumor diagnosis.

### Carbon Quantum Dots

Carbon quantum dots (CQDs), commonly known as carbon dots (CDs), are quasi-spherical fluorescent particles with sizes <10 nm. Compared with GQDs, CQDs have poorer crystallinity, which is due to the lower content of crystalline sp^2^ carbon and more surface defects (Pirzada and Altintas, 2019). CQDs possess excellent optical properties in fluorescence, chemiluminescence (CL) and electrochemiluminescence (ECL); therefore are widely used in fields of bioimaging, drug delivery and biosensing (Atabaev, 2018; Molaei, 2019b). Similar to GQDs, CQDs can be synthesized and functionalized quickly and easily. The doping or surface functionalization can further improve the topical chemical properties, optical properties, surface reaction activity and biocompatibility of CQDs, so as to improve their sensitivity as biosensors (Molaei, 2019a). In this part, recent advances of CQDs in ion detection and disease diagnosis are reviewed in detail.

**Figure 2:**
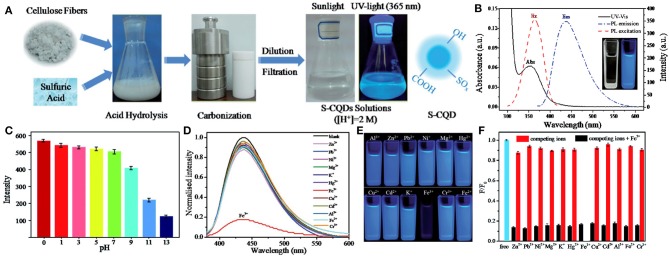
**(A)** Synthesis procedure of S-CQDs. **(B)** UV-vis absorption (Abs), PL excitation (Ex, λ_em_ 435 nm) and emission spectrum (Em, λ_ex_ 360 nm) of S-CQDs in pH 0 aqueous solutions. The inset shows the photograph of the S-CQDs solution under daylight (left) and UV light (right). **(C)** PL intensity of S-CQDs in different pH solutions. **(D)** PL spectrum of S-CQDs in pH 0 aqueous solutions with 2 mM of different metal ions. **(E)** Corresponding photographs of different metal ions. **(F)** Relative PL intensities (F/F0) of S-CQDs in pH 0 aqueous solutions with 2 mM of various metal ions (red bar) and after treating with 2 mM Fe^3+^ (black bar). Reproduced with permission from Yang G. et al. (2016). Copyright 2016, Royal Society of Chemistry.

The use of CQDs in metal ion sensing has been developing rapidly, and a large number of CQDs-based electrochemical and fluorescent sensors have been reported. For instance, Fan et al. constructed a functionalized CQDs-modified gating electrode for Cu^2+^ detection based on solution-gated graphene transistors. The combination of CQDs and Cu^2+^ led to the change of the capacitance of the double electrical layer near the gate electrode, which further led to the change of channel current. Since Cu^2+^ bounded with CQDs very well, the detection limit of Cu^2+^ in the CQDs-modified sensor was as low as 1 × 10^−14^ M (Fan et al., 2020). Another example of ion detection is the application of CQDs in the rapid quantitative detection of heavy metal contamination in water. Yang et al. synthesized a novel S-doped CQDs (S-CQDs) with excellent selectivity and sensitivity for the detection of Fe^3+^ in pH 0 solutions (Figure 2). These S-CQDs exhibited strong acidophilia, high luminescence and high quantum yield (up to 32%) in strong acid solutions, and the detection limit for Fe^3+^ was as low as 0.96 μM. S-CQDs also had robust stability. After 8 cycles, these pH-switch PL properties of S-CQDs were not significantly affected. These results showed that doped CQDs have a promising prospect in the detection of heavy metal pollutants in strong acid environments (Yang G. et al., 2016).

In addition, CQDs can also be utilized to detect intracellular biomolecules (Loo et al., 2016; Liu T. et al., 2017; Zhang Q. Q. et al., 2018). Selenoproteins are involved in a variety of cellular functions and are associated with various human diseases such as cancer and cardiovascular diseases. Wang et al. prepared a CQDs-based novel fluorescent nanoprobe for the fluorescence imaging of selenol *in vitro*. After selenocysteine treatment, the 2,4-dinitrobenzenesulfonyl chloride fragment of CQDs was split easily by selenolate to form yellow-green fluorescence CQDs. The prepared nanoprobe was highly sensitive and selective to selenol and could achieve the fluorescence imaging of exogenous and endogenous selenol in living cells. By adding different identifying elements, the functionalized CQDs were expected to be used to detect other biological analytes (Wang et al., 2017).

Besides, CQDs have been successfully used in cancer diagnosis. Lu et al. developed an ultra-sensitive fluorescent sensor platform based on nitrogen-doped CQDs (N-CQDs) for the detection of tumor invasive biomarker β-glucuronidase (GLU). In this sensor platform, N-CQDs with green PL were used as the fluorophore, and 4-nitrophenyl-catalyzed d-glucuronide served as the GLU substrate. The GLU catalytic product (p-nitrophenol) acted as a robust absorber to turn off the fluorescence of N-CQDs. Therefore, the activity of GLU could be reflected by the fluorescence intensity of N-CQDs. The as-prepared sensor could detect GLU with high sensitivity and the detection limit was 0.3 U/L. This sensing strategy avoided complex modification of fluorophores or covalent bond connections between receptors and fluorophores; therefore provided a new idea for the development of sensitive sensors to detect tumor biomarkers by fluorescent CQDs (Lu et al., 2016).

### Fullerenes

Fullerene is a molecular allotrope of carbon discovered in 1985 by Kroto et al. C_60_, the most common fullerene, consists of five to six sp^2^ hybrid carbon rings, forming a truncated icosahedron (Carneiro et al., 2019; Pirzada and Altintas, 2019). Fullerenes exhibit high electron affinity, large surface volume ratios and structural stability (Sun et al., 2018). With these characteristics, fullerenes are used in a diverse range of applications, including electronics, biology and medicine. In addition, fullerenes also possess good biocompatibility and inertia, and have good affinity with various organic molecules; therefore can be used to construct various biosensors (Winkler et al., 2006; Afreen et al., 2015). As a nanomaterial for amplifying detection signals, fullerenes are also used in the ultra-sensitive detection of analytes in different chemical and biological materials, such as amino acids, DNA and biomarkers for early-stage cancer diagnosis (Miyazawa, 2015).

Because of their large electroactive surface area, fullerenes are often used to construct electrochemical biosensors to detect amino acids. As an example, Jaiswal et al. produced a C_60_-based electrochemical sensor to quantitatively distinguish D- from L- serine, which was essential to the function of central nervous system. This sensor demonstrated a very good analytical performance, showing a detection limit of 0.24 ng/mL for both isomerides. The experimental results showed that the C_60_-based electrochemical sensor could maintain the original performance without current deviation, and was durable in water samples for up to 3 weeks. In view of this, this sensor had higher detection sensitivity, stability and repeatability than previously reported sensors. It could be a promising tool for the diagnosis of schizophrenia in clinical patients (Jaiswal et al., 2019).

**Figure 3:**
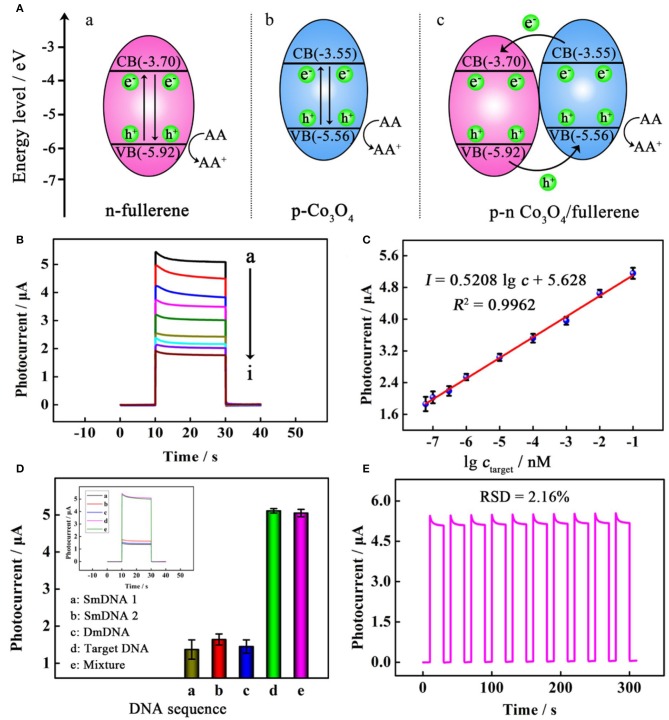
**(A)** Schematic illustration of the proposed mechanism for the photocurrent responses of (a) n-fullerene, (b) p-Co_3_O_4_, and (c) p–n Co_3_O_4_/fullerene. (B) Photocurrent responses of the PEC biosensor toward a target at different concentrations (from top to bottom): (a) 100 pM, (b) 10 pM, (c) 1 pM, (d) 100 fM, (e) 10 fM, (f) 1 fM, (g) 0.3 fM, (h) 0.1 fM, and (i) 0.06 fM. **(C)** linear relationship between photocurrent responses and the logarithm of the target concentration. **(D)** selectivity with photocurrent responses in the inset for incubating different samples; and **(E)** stability of the PEC biosensor. Reproduced with permission from Wang H. H. et al. (2019). Copyright 2019, American Chemical Society.

In another study, a photoelectrochemical (PEC) biosensor based on fullerenes was prepared to detect DNA. Wang et al. synthesized a smart PEC biosensor based on Co_3_O_4_-fullerene to realize ultrasensitive DNA detection, in which p–n-sensitized heterostructure Co_3_O_4_-fullerene as an efficient sensitizer improved the photoelectric conversion efficiency greatly, making it 6 times higher than that of the fullerene alone (Figure 3). As reported, the Co_3_O_4_-fullerene biosensing structure was successfully used in the ultrasensitive investigation of model DNA (a fragment of the p53 gene) with a detection limit of 20 aM and a wide linear range from 60 to 1 × 10^5^ aM (Wang H. H. et al., 2019). This study opens a fascinating avenue to construct sensitized photoconductive candidates with excellent PEC performance and exhibits significant application foreground in the detection of biomolecules.

Like GQDs and CQDs, fullerenes also have their use in cancer diagnosis. For example, Yuan et al. constructed a sandwich-type electrochemical biosensor based on 4-MPBA@n-C_60_-PdPt to detect tumor biomarkers α2,3-sialylated glycans. Maackia amurensis lectin (MAL) was fixed on Au-poly (Au-PMB) to form a specific recognition tool for α2,3-sial-Gs. Amino-functionalized fullerene (n-C_60_) was introduced to the surface of PdPt bimetallic alloy to further improve the load capacity and conductivity of the sensor. The n-C_60_ nanomaterial had a good electron transferability, which could accelerate the electron transfer rate and improve the sensitivity of the biosensor. The detection limit was observed to be as low as 3 fg/mL (S/N = 3). Moreover, this sensing platform exhibited good recovery and stability, indicating its potential application in clinical research (Yuan et al., 2018).

In conclusion, we have discussed three kinds of 0D carbon-based nanomaterials used in different sensors such as PL sensor, electronic sensor, electrochemical sensor and electrochemiluminescence sensor that can be applied to ion detection, biomolecular recognition and disease diagnosis (Sun et al., 2013; Raja et al., 2019; Dhenadhayalan et al., 2020). Although 0D carbon-based nanomaterials have been noticed by a lot of researchers, research on them is still in the initial stage. Current synthesis methods cannot control the structure of materials at the atomic level. Moreover, the fluorescence quenching mechanism of 0D carbon nanomaterials has not been well-explained. In the future, the synthesis accuracy and optical and electrical properties of 0D carbon-based nanomaterials should be studied, so as to prepare biosensors with more clinical application value.

## Quantum Dots

As fluorescent semiconductor nanocrystals, quantum dots (QDs) are generally prepared with atoms from group II-VI or III-V in the periodic table. The most common QDs, such as CdTe, CdSe, and InP, have potential applications in various biomedicine fields, including bioimaging, biosensing, and therapy (Wegner and Hildebrandt, 2015; Xiao et al., 2019). Because of the toxicity of heavy metals, the biocompatibility of inorganic QDs is generally questioned. To some extent, this problem is solved by the synthesis of QDs in aqueous solution, which improves not only the biocompatibility but also the water solubility and stability. Emerging heavy metal-free QDs such as SiQDs can also be an ideal alternative to commercially available cytotoxic CdTe and CdSe QDs (Keshavarz et al., 2018). QDs have been used to develop a variety of fluorescence, chemiluminescence and bioluminescence sensors due to their unique optical properties such as enhanced brightness, resistance to photobleaching, large absorption coefficient, narrow emission spectrum and size-tunable light emission (Liang et al., 2014b; Ma et al., 2019). In this section, we will focus on the application of QDs in biosensing in recent years.

Owning to their unique photochemical stability and high PL quantum yields, QDs have been widely employed in pathogen detection. For example, Zhang et al. functionalized an integrated microfluidic chip with high-luminance QDs and magnetic nanoparticles for the detection and subtyping of multiple influenza viruses (H9N2, H3N2, H1N1) simultaneously. By conducting superparamagnetic beads and QDs-assisted multiple DNA hybridization detection on microfluidic chips with controlled micro-magnetic fields, H9N2, H3N2, and H1N1 cDNAs could be simultaneously detected within 80 min with a very low sample and reagent consumption (only 3 μL). This was a convenient, time-saving, cost-effective, and highly sensitive way; therefore could be a powerful technology platform for the rapid detection of multiple influenza viruses (Zhang R. Q. et al., 2018).

In addition, water-soluble and low toxic QDs can be used for cell detection and dynamic evaluation. As a paradigm, Li et al. constructed a novel PEC biosensing platform combining near-infrared (NIR) Ag_2_S QDs with AuNPs for the non-destructive analysis of living cells. The water-dispersed Ag_2_S QDs were photoelectrochemically active species with excellent PEC properties under NIR. Under NIR light of 810 nm, the linear range in this strategy was 1 × 10^2^ to 1 × 10^7^ cells/mL, and the detection limit was 100 cells/mL (Li et al., 2018c). All these experimental results show that NIR QDs have a good application prospect in the construction of novel PEC platforms for the detection of biomolecules.

**Figure 4:**
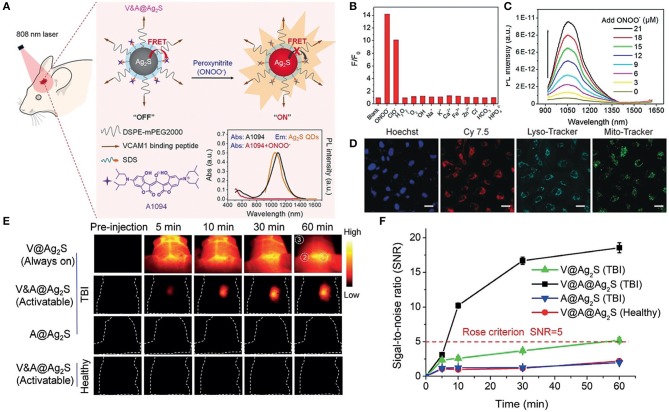
**(A)** Schematic diagrams of the synthesis procedure of the V&A@Ag_2_S probe and detection of ONOO^−^ *in vivo*. **(B)** NIR-II fluorescence intensity changes of V&A@Ag_2_S upon the addition of various ROS/RNS analytes and ions. **(C)** Photoluminescence recovery of V&A@Ag_2_S in aqueous solutions as a function of ONOO^−^ concentration. **(D)** Confocal fluorescence images of inflamed endothelial cells. The cells were incubated with Cy7.5-modified V&A@Ag_2_S first and then further incubated with LysoTracker, MitoTracker, and Hoechst. **(E)** The timespan of NIR-II fluorescence in brain vascular injury and healthy mice at different time points after injection of V@Ag_2_S, V&A@Ag_2_S, and A@Ag_2_S. **(F)** Time-dependent signal-to-noise ratio (SNR) changes determined by the NIR-II fluorescence imaging of mice after various treatments. Reproduced with permission from Li et al. (2020). Copyright 2020, Wiley-VCH.

Moreover, QDs-based biosensors can be employed as universal tools to detect biomarkers of cancer and brain diseases. For instance, Li et al. designed a new kind of ECL biosensor based on CdSe@ZnS QDs that combined target recovery amplification with a double-output conversion strategy to achieve the ultrasensitive detection of microRNA in human prostate cancer cells. Experimental results showed that the ECL biosensor could quantitatively detect miRNA-141 in a wide range from 100 aM to 10 pM with a detection limit of 33 aM (Li et al., 2017). In another work, Li et al. prepared a V&A@Ag_2_S QDs fluorescent nanoprobe in the second near-infrared (NIR-II) window for the real-time *in vivo* imaging of early biomarkers of traumatic brain injury (TBI) (Figure 4). The fluorescence of Ag_2_S was turned off due to the energy transfer from Ag_2_S to A1094 chromophore. After intravenous injection, A1094 was bleached by the TBI precursor biomarker peroxynitrite (ONOO^−^), achieving rapid recovery of Ag_2_S QDs fluorescence. This NIR-II *in vivo* turn-on sensing and imaging strategy indicated a broad prospect of QDs fluorescence imaging in clinical applications (Li et al., 2020).

SiQDs are a new type of heavy metal-free QDs developed in recent years. They have the advantages of aqueous solubility, low cost, high quantum yield and strong resistance to photobleaching. Because of these interesting properties and good biocompatibility, SiQDs are widely used as fluorescent probes to detect small molecules, ions and biological macromolecules. Li et al. prepared SiQDs through a one-pot approach by using 3-(aminopropyl)-trimethoxysilane (APTMS) as precursors. The incorporation of salicylaldehyde effectively inhibited SiQDs emission through nucleophilic reaction. In addition, the addition of Zn^2+^ led to the evolution of emission peak. The green band at 500 nm gradually shifted to the blue direction at 455 nm, and corresponding changes in the ratiometric signal (I_455_ / I_500_) could accurately reflect the concentration of Zn^2+^, with a detection limit of 0.17 μM (Li et al., 2018d). These fluorescence emission changes based on SiQDs would provide a new idea for the development of nanoscale functional sensors.

In general, we have introduced various biosensing applications of QDs in this part, which greatly improves the comprehension of QDs as potential biomaterials for biosensing applications (Wegner and Hildebrandt, 2015; Ma et al., 2019). QDs have a good affinity for biomolecules and their chemical and optical properties enable their use as optical sensors to detect various biomolecules (Yao et al., 2018). It is expected that more QDs-based research in fields of pathogen detection, non-destructive analysis of living cells and disease diagnosis will be conducted.

## Magnetic Nanoparticles

0D magnetic nanoparticles (MNPs), with a size range of 1-100 nanometers, are composed of materials with high saturation magnetization, such as pure metals (Fe, Co, Ni), alloys (FeCo, permalloy, alnico) and oxides (Fe_3_O_4_, CoFe_2_O_4_) (Lin et al., 2017; Gloag et al., 2019). MNPs have attracted extensive attention in hyperthermia treatment, biosensing and drug delivery due to their high saturation magnetization and superparamagnetism (Nabaei et al., 2018). Biosensors with 0D MNPs are important in the field of sensing because they provide viable solutions to the long-term challenge of low detection limits and non-specific effects (Morón et al., 2015). Modulation of the size, composition and magnetic properties of MNPs benefits their application in the ultralow detection of proteins, disease biomarkers and pathogens (Chen Y. et al., 2018; Knežević et al., 2019).

**Figure 5:**
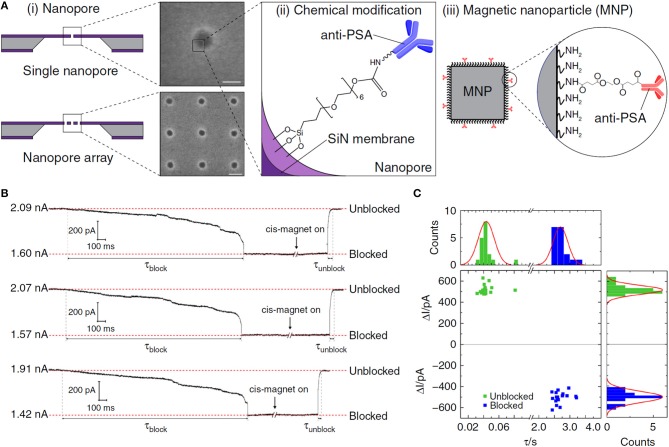
**(A)** (i) Schematics and scanning electron micrographs of (top) a solid-state nanopore; (bottom) a nanopore array in SiN membrane. (ii) Illustration of a chemically modified SiN nanopore with silane-EG_6_-(anti-PSA) self-assembled monolayer (SAM). (iii) Illustration of an (anti-PSA)-conjugated MNP used in this work. Inset: chemical structure of the (anti-PSA)-EG_6_ immobilized onto the amine-rich PEI coating of the MNP. **(B)** Controlled blocking and unblocking of nanopore with MNP. Concatenated ionic current traces showing blocking and unblocking events of a 27 nm solid-state nanopore modified with silane-EG_6_ SAM by 50 nm MNPs. **(C)** Nanopore blocking and unblocking event statistics. Each data point in the scatter plot corresponds to the parameters of individual blocking and unblocking events (*n* = 18, respectively) measured from ionic current traces. Reproduced with permission from Chuah et al. (2019). Copyright 2019, Nature Publishing Group.

MNPs can be used for protein capture after combined with specific recognition molecules. For example, Chuah et al. synthesized anti-prostate-specific antigen (PSA)-labeled MNPs to selectively capture protein analyte PSA (Figure 5). In the external magnetic field, the (anti-PSA)-MNPs captured PSA and rapidly carried them into nanopores. After (anti-PSA)-MNPs captured PSA, they would form a sandwich complex with the anti-PSA antibodies in the nanopore. The magnetic field was then reversed to remove the (anti-PSA)-MNPs that did not capture PSA to avoid miscounting. The detection limit of this nanopore sensor was 0.8 fM, which was nearly an order of magnitude higher than that of the previous nanopore sensors. This MNPs nanopore blockade concept could be further expanded to other proteins with suitable antibodies (Chuah et al., 2019).

MNPs have exhibited tremendous potential in early-stage cancer diagnosis. The ability of biosensors to detect ultralow levels of circulating microRNAs in the blood is significant for the development of liquid biopsies to monitor the progression of diseases. Tavallaie et al. synthesized a network of gold-coated MNPs modified by probe DNA (DNA Au@MNPs) to directly analyze nucleic acids in whole blood. The sensor for the first time detected the concentration of microRNA in untreated blood samples (10 aM to 1 nM). *In vivo* experiments showed that it could monitor the small changes in microRNA concentration in the blood of tumor-growing mice. The use of electrically reconfigurable DNA Au@MNPs network to supersensitively and directly detect microRNAs made the device a promising tool for cancer diagnosis (Tavallaie et al., 2018). In another study, Pal et al. designed an MNPs-Abs-based fluorescence spectroscopic platform to analyze ovarian cancer biomarkers [cancer antigen 125 (CA-125), β2-microglobulin (β2-M), and Apolipoprotein A1 (ApoA1)]. A sandwich method was established with the help of polyclonal antibodies. Sandwich particles were extracted from the sensing medium by magnetic force, and fluorescence changes at standard concentrations were monitored in real time. The detection limits were 7.7 ng/mL, 0.55 ng/mL and 0.26 U/mL respectively. The sensor also successfully distinguished ovarian cancer patients from healthy individuals with a sensitivity of 94% and a specificity of 98% (Pal et al., 2015).

MNPs-based biosensors have also been studied for the diagnosis of other diseases. Lee et al. constructed functionalized Au@Fe_3_O_4_ core-shell structures to detect asthma biomarker eosinophil cationic protein (ECP). The core-shell magnetic nanostructures modified by cysteamine-tagged heparin (Hep) amplified the differences in electrochemical signals, thus increasing the sensitivity of the biosensor. This method provided a wide linear range of 1~1000 nM for the logarithmic analysis of ECP concentrations, with a determination coefficient of 0.992 and a detection limit of 0.30 nM. It could be applied to the sensitive detection of other analytes by replacing the Hep/ECP pair with a relevant probe/target combination (Lee et al., 2018).

The unique optical properties and magnetism of MNPs can be used to construct biosensors for pathogen detection. Alhogail et al. fabricated a low-cost colorimetric biosensor based on MNPs for the rapid clinical detection of pseudomonas aeruginosa. The detection limit was as low as 10^2^ cfu/mL within one min. This biosensor is expected to be a rapid medical device to diagnose pseudomonas aeruginosa-related infections (Alhogail et al., 2019). As we know, Norovirus (NoV) can cause infectious diarrhea which is highly contagious and can spread quickly. Takemura et al. developed an AuNP/MNP hybrid nanocomposite for the hypersensitive detection of NoV. The high localized surface plasmon resonance (LSPR) effect was achieved by combining the AuNP/MNP hybrid nanocomposite with CdSeS QDs using anti-norovirus genome II antibody (Ab). This sensor system could be applied to norovirus detection in fecal samples with a detection limit of 0.48 pg/mL (Takemura et al., 2019).

To sum up, remarkable progress has been made in the application of MNPs to single-molecule detection, disease diagnosis as well as pathogen detection. In biosensing devices, MNPs can be assembled onto the sensor surface or used as labels (Weddemann et al., 2010; Chen Y. et al., 2018). In addition to the structure and composition of MNPs, the selection of surface functional groups and target molecules is also crucial to the expansion of MNPs to a variety of biosensor applications (Chen et al., 2017; Avval et al., 2019). Moreover, the stability of MNPs is particularly important in biological systems where there are many oxidizing and reducing substances. A stable coating of the functionalized MNPs is essential to ensure that the MNPs do not accumulate or cause changes in their finely controlled magnetic properties. Therefore, adjusting the composition of MNPs and immobilizing appropriate functional groups on the surface play important roles in significantly improving the sensitivity and stability of biosensor pieces.

## Noble Metal Nanoparticles

Noble metal nanoparticles are a kind of nanoscale ultrafine particles with totally different properties from macroscopic metals. They have attracted more and more attention due to their small sizes, good biocompatibility and excellent photophysical properties. In the presence of light, nanoparticles can generate electron resonance; therefore the scattering and absorption of light can be easily enhanced (Chen J. B. et al., 2019; Zhao X. et al., 2019). Noble metal (gold, silver, etc.) nanoparticles as fluorescent probes have a broad application prospect in biomedical fields (Kim et al., 2017; Wang H. et al., 2019; Zhao Y. et al., 2019). Traditional organic fluorescent dyes often have the disadvantages of fast photobleaching rate, short fluorescence life and high biotoxicity, which can be overcome by the unique optical thermoelectricity and biocompatibility of noble metal nanoparticles (Koo Lee et al., 2009). In this section, we will focus on the application of noble metal nanoparticles as biosensors in biomolecular detection and disease diagnosis.

### Gold Nanoparticles

Gold nanoparticles (AuNPs) are conductive materials with a large surface area and unique optical properties (Kurochkina et al., 2018). In AuNPs, the surface plasma was constrained to produce high LSPR (Pirzada and Altintas, 2019). By adjusting the size, shape and polymerization of AuNPs, it is possible to develop multifunctional AuNPs that can be used as optical and electrical biosensors (de la Escosura-Muñiz et al., 2016; Tian et al., 2016; Mei et al., 2018). Currently, functional AuNPs used for detecting biomarkers for cancer, neurological diseases, proteins, nucleic acids, and various pathogens are extensively studied.

The excellent electrical conductivity of AuNPs can enhance the electron transfer between the redox center and the electrode surface, providing abundant active sites for the highly sensitive detection of cancer biomarkers by the biosensor electrode (Altintas et al., 2014; Yan et al., 2015; Jia et al., 2017). For instance, Tran et al. used AuNPs to develop a bimetallic nanocrystal platform for the highly sensitive detection of carcinoembryonic antigen (CEA). Since the combination of Au and Cu enhanced the active surface area of Au and enhanced the interaction between these two components, this nanohybrid-based biosensor showed excellent electrochemical sensitivity to CEA detection with a very low detection limit (0.5 pg/mL), proving that the AuNPs-based sensor had potential application in the diagnosis of tumors (Tran et al., 2018).

**Figure 6:**
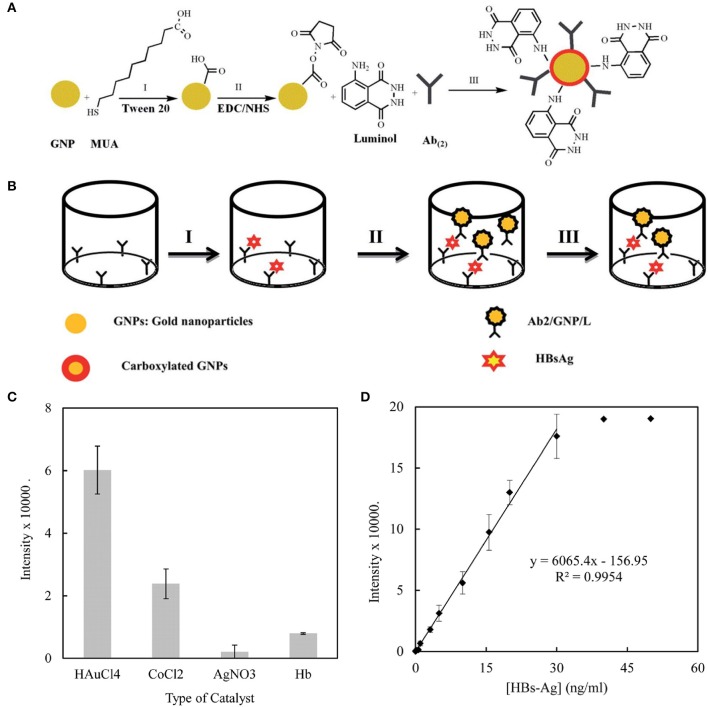
**(A)** The process for the functionalization of the gold nanoparticles. **(B)** The process for the immune sandwich formation and chemiluminescence measurement. **(C)** A comparison of the effects of different catalysts on the immunoassay. The chemiluminescence intensity of the sandwich containing HBsAg (10 ng/mL) was recorded in the presence of H_2_O_2_ (10^−3^ M) and different catalysts at their optimized concentration: AgNO_3_ 10^−5^ M, CoCl_2_ 0.01 M, HAuCl_4_ 0.1% w/v, Hb (150 mg/mL) in SCB (100 mM, pH 9). **(D)** The calibration curve of the immunosensor toward HBsAg. Reproduced with permission from Sabouri et al. (2014). Copyright 2014, Royal Society of Chemistry.

Various clinically relevant compounds, including neurotransmitters and antigen, have been detected using AuNPs. Parkinson's disease (PD) is a common neurodegenerative disease whose standard treatment is levodopa supplementation. However, excess accumulation of levodopa can lead to dyskinesia and emotional incontinence. Therefore, Ji et al. developed an AuNPs-based electrochemical detection system to monitor levodopa concentration in real time. In this system, AuNPs were used to modify the electrode to improve the electrical conductivity and electrocatalytic performance so as to increase the sensitivity of the sensor. This sensor could detect levodopa at concentration as low as 0.5 μM in human serum (Ji et al., 2019). In another study, AuNPs were used to detect hepatitis B surface antigen. Sabouri et al. designed a gold nanoparticle-based immunosensor to detect HBsAg with a linear concentration range of 0.12 ~ 30 ng/mL and a detection limit of 14 pg/mL (Figure 6). The prepared immunosensor possessed the advantages of low cost, easy-to-operate and time-saving, and was expected to be used in clinical immunoassay (Sabouri et al., 2014).

AuNPs are also frequently used in pathogen detection (Altintas et al., 2018; Savas et al., 2018; Zhang X. et al., 2019). Outbreaks of zika virus (ZIKV) in the tropics have posed major challenges to global health in recent years. Steinmetz et al. constructed a novel impedance DNA biosensor based on an oxidized glassy carbon electrode. Due to the high surface area and high conductivity of AuNPs, the payload of the DNA probe increased obviously and the electrochemical response sensitivity of the developed biosensor was significantly improved. The detection limit of the device was determined to be 0.82 pM by electrochemical impedance spectroscopy (EIS). After 90 days of evaluation with EIS, the response of the sensor was about 98.0% of the initial response, indicating the good stability of the biosensor. This the device was expected to be used as a commercially viable diagnostic tool for ZIKV (Steinmetz et al., 2019). Furthermore, AuNPs can be used to construct fluorescent sensors for pathogen detection due to their unique optical properties. Lee et al. used gold nanoparticle-decorated carbon nanotubes to construct a plasmon-assisted fluoro-immunoassay platform to detect influenza viruses. The minimum detection limit for the influenza virus was 0.1 pg/mL. This fluorescence immunoassay system also had good selectivity to influenza virus, which was 100 times higher than that of commercial diagnostic kits (Lee et al., 2015).

### Silver Nanoparticles

Silver nanoparticles (AgNPs) have similar physical and chemical properties to gold nanoparticles and are commonly used in medical diagnosis. Due to the LSPR absorption, the optical properties of AgNPs are also influenced by their shape, size and degree of aggregation (Chen J. B. et al., 2019; Xu H. V. et al., 2019). However, AgNPs have fewer applications than AuNPs due to the concern about cytotoxicity. Nevertheless, the use of AgNPs in biomedical research is increasingly encouraged because of their antifungal and antibacterial properties (Malekzad et al., 2017; Pirzada and Altintas, 2019). In addition, AgNPs have attractive electrical properties. Compared with AuNPs of the same dimension, they have higher extinction coefficient and are more prone to electrochemical oxidation (Bahrami et al., 2016). In the field of biosensing, AgNPs have been widely used in SERS-based biosensors to improve their performance due to their strong plasmon resonance characteristics.

**Figure 7:**
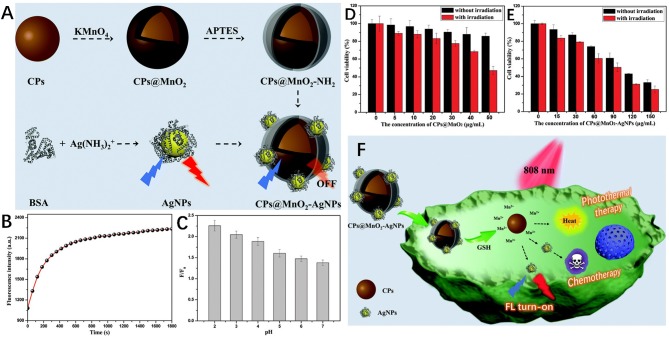
**(A)** Schematic diagrams of the synthesis of CPs@MnO_2_, AgNPs and the CPs@MnO_2_-AgNP nanocomposite. **(B)** The fluorescence intensity of CPs@MnO_2_-AgNPs changes with time in the presence of GSH. **(C)** The pH-dependent fluorescence recovery efficiency of CPs@MnO_2_-AgNPs in the presence of GSH. Viability of SMMC-7721 cells after incubation with different concentrations of **(D)** CPs@MnO_2_, and **(E)** the CPs@MnO_2_-AgNP (irradiated or not). **(F)** Schematic diagram of fluorescence recognition and combined therapy of cancer by the CPs@MnO_2_-AgNP nanocomposite. Reproduced with permission from Wang Q. et al. (2019). Copyright 2019, Royal Society of Chemistry.

AgNPs-based nanocomposites have excellent recognition ability and specific response in tumor microenvironment; therefore can be used for the biomolecular sensing of cancer cells and cancer treatment. A notable feature of tumor microenvironment is that the GSH level is significantly higher than that of normal tissues (Gao et al., 2018; Peng et al., 2018). Wang et al. constructed a multifunctional nanosensor of carbon nanoparticles (CPs)@MnO_2_-AgNPs for GSH sensing and cancer diagnosis (Figure 7). In this sensor, the AgNPs fluorescence was quenched by MnO_2_ through internal filter effect and static quenching effect, and then recovered by GSH due to MnO_2_ decomposition. The detection limit of GSH was as low as 0.55 μM. In addition, due to the photothermal activity of CPs and the chemotherapy effect of AgNPs as anti-proliferators, the prepared multifunctional nanocomposite could be used for cancer fluorescence identification and combination therapy (Wang Q. et al., 2019).

AgNPs have also been used to test various drugs and monitor their effects on human body. Recently, a sensitive biosensor based on AgNPs and electrochemical reduced graphene oxide nanocomposites (AgNPs: ErGO/PG) was developed to determine the effect of caffeine (CAF) on estradiol (EST) concentration in women of childbearing age (18–35 years). According to the analysis of EST and CAF in serum and urine samples of 5 women of child-bearing age, the detection limits of EST and CAF were 0.046 and 0.54 nM, respectively (Raj and Goyal, 2019). Mao et al. synthesized Au@Ag core-shell nanoparticles for the SERS detection of methylamphetamine (MAMP). Compared with AuNPs, SERS with a core-shell structure had better performance. By adjusting the concentration of MAMP adaptor modified on the surface of the sensing platform, the highly sensitive detection of MAMP was realized. The MAMP sensor had a wide dynamic range of 0.5 ppb to 40 ppb and a detection limit of 0.16 ppb. All these results indicated that AgNPs could be used for the rapid screening of abused illegal drugs and the detection of drug content *in vivo* (Mao et al., 2018).

AgNPs are one of the most widely used antibacterial nanomaterials. Apart from their use as antimicrobial agents, AgNPs are also broadly used in the construction of electrical analysis platforms due to their unique functions such as enhanced electron transfer and controlled electrode microenvironment (Zheng et al., 2018; Hussain et al., 2019). Yang et al. prepared vancomycin-functionalized AgNPs/3D-ZnO nanorod arrays for the detection and clearance of pathogenic bacteria. Based on Van's specific identification of gram-positive bacteria, the constructed electrochemical platform was highly sensitive to the detection of staphylococcus aureus with a detection limit of 330 cfu/mL. Furthermore, the platform had a high antibacterial activity (99.99%) due to the synergistic bactericidal effect of AgNPs and Van (Yang et al., 2017).

In this section, the recent development of AuNPs and AgNPs in biosensing fields including the detection of cancer markers, pathogens and drug analytes has been summarized and discussed. Despite the remarkable progress, the clinical application of noble metal nanoparticles still faces great challenges. In order to prepare multifunctional noble metal nanoparticles with excellent optical and electrical properties, their synthesis and modification methods need to be optimized (Guo and Wang, 2011; Zhao X. et al., 2019). To improve sensor specificity, the discovery of functional molecules is very crucial. The selectivity of noble metal nanoparticles can be effectively improved through functionalization (Chen J. B. et al., 2019; Pirzada and Altintas, 2019; Wang H. et al., 2019). In addition, the correlations between the composition, structure and performance of noble metal nanoparticles sensors needs to be further studied.

## Other Types of 0D Nanomaterials

In addition to the above 0D nanomaterials, UCNPs and Pdots also have their application in biosensing. UCNPs have attracted extensive attention for their highly efficient up-conversion PL and photobleaching resistance, and their cytotoxicity is much lower than that of other nanoparticles (Park et al., 2015). As a type of fluorescent nanoparticles, Pdots have attracted much interest because of their good photostability, high brightness, and facile surface functionalization of fluorescence (Cheng X. et al., 2019). In the following part, the biosensing application of these three 0D nanomaterials will be introduced.

### Upconversion Nanoparticles

UCNPs are a special class of lanthanide-doped nanoparticles capable of converting NIR light into multicolor and high-energy ultraviolet/visible light. As an efficient fluorescence resonance energy transfer (FRET) donor, UCNPs can be used to construct FRET sensor platforms for the detection of various biomolecules (Liu C. et al., 2019; Wang F. et al., 2019). For example, Liu et al. synthesized a FRET sensing platform based on peptide-functionalized UCNPs for the specific detection of apoptosis-associated caspase-9 activity *in vitro* and *in vivo*. Examination of caspase-9 activity *in vitro* showed that the UCNPs-based sensing platform could identify cisplatin mediated changes in intracellular caspase-9 activity with a detection limit of 0.068 U/mL. Moreover, due to its excellent FRET performance, low cytotoxicity and good colloidal stability, this UCNPs-based sensing platform was successfully used *in vivo* to visualize caspase-9 activity. Such a UCNPs-based biosensor could serve as a powerful sensing platform to monitor the apoptotic process and evaluate anti-cancer drug efficacy (Liu L. et al., 2019).

**Figure 8:**
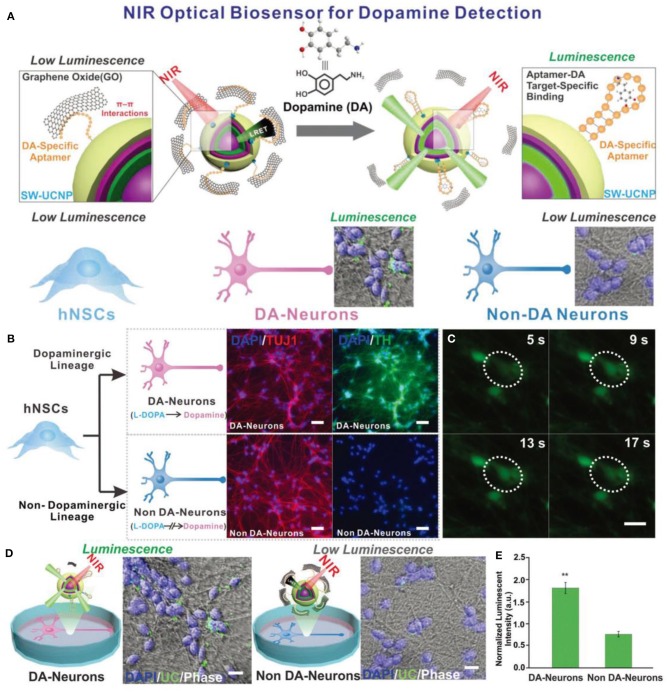
**(A)** Schematic diagrams of the constructed NIR-based dopamine sensor and its application in live cell dopamine sensing in non-DA-neurons and DA-neurons. **(B)** Immuno-fluorescence imaging of DA-neurons and non-DA-neurons. (Blue: Nucleus (DAPI). Red: β tubulin (TUJ1). Green: Tyrosine Hydroxylase (TH).) **(C)** Spontaneous calcium fluctuations determined by Fluo4 fluorescence for an active DA-neuron (white circle). **(D)** Live cell upconversion luminescence imaging with DA and non-DA-neurons. **(E)** Live cell DA sensing quantitative comparison through microscope imaging signal. Reproduced with permission from Rabie et al. (2019). Copyright 2019, Wiley-VCH.

In addition, like GQDs and AuNPs, UCNPs can also be used for neurotransmitter sensing. Rabie et al. developed a Yb@Er@Yb sandwich UCNPs sensing platform with a wide detection range from 1 pM to 10 pM to detect dopaminergic neurons (DA) in stem cell-derived neural interface (Figure 8). The as-prepared UCNPs-based sensor platform overcame the disadvantage of low emission intensity of traditional UCNPs, and generated bright visible light emissions in response to low power density NIR excitation. The highly efficient upconversion process of UCNPs greatly improved the detection sensitivity to DA. Therefore, this UNCPs-based biosensor could be used to detect neurotransmitters in stem cell-derived neural interface, showing great potential in the neuroscience and stem cell biology fields (Rabie et al., 2019).

### Polymer Dots

As advanced nanomaterials, Pdots generally possess high fluorescence brightness and high photostability (Alizadeh and Salimi, 2019; Kim et al., 2020). In recent years, Pdots have emerged as interesting fluorescent probes in biosensors and are widely used in biomolecular detection. Luo et al. designed a coreactant-free dual amplified ECL sensing platform with conjugated Pdots as luminophores for the hypersensitive detection of miRNA. Conjugated Pdots with high carrier mobility could obtain a super-strong ECL signal without adding any coreactant, thus improving the sensitivity of the sensor. As a result, this sensing platform showed a minimum detection limit of 3.3 aM for RNA. These conjugated Pdots provided a good platform for the construction of coreactant-free ECL biosensors and expand the application of Pdots in the clinical analysis (Luo et al., 2019).

In general, the above mentioned emerging 0D nanomaterials (UCNPs, Pdots, SiQDs) have made important contributions to ion detection and biomolecular detection in the field of biosensing. However, experimental studies on their application in disease diagnosis remain rare, and there is also short of studies on their metabolism, biodegradation and long-term toxicology. What's more, emphasis should be attached to the innovation of synthesis methods and realization of large-scale preparation. It is also worth noting that the functional modification can also benefit the application of these 0D nanomaterials in the field of biosensing.

## Conclusions and Perspectives

0D nanomaterials are characterized by small volume, high surface-to-volume ratio, edge and quantum constraint effect and good biocompatibility. Due to the unique structures and properties, the application of 0D nanomaterials in the field of biosensing is expanding rapidly. In this short review, we have highlighted recent progress in 0D nanomaterials regarding their structures, properties and biosensing applications. Carbon-based nanomaterials (GQDs, CQDs, fullerenes), inorganic QDs, MNPs and noble metal nanoparticles (AuNPs, AgNPs) remain to be the research focuses in the biosensing field. The recently studied UCNPs, Pdots, and SiQDs exhibit promising application prospects. However, the research of all these nanomaterials in biosensing is still at the immature stage. In order to advance this field and promote further clinical application, several challenges need to be addressed.

Although the emergence of various fabrication methods has made the synthesis of intricately tuned 0D nanomaterials possible, no specific atomic precise structure has been reported, which limits the in-depth study of the relationship between the structure and performance, the precise control of performance and the sensing mechanism. For instance, a great deal of research has been conducted on the sensing of metal ions. Most of them involve the fluorescence quenching of 0D nanomaterials, but how such quenching is carried out and to what extent the selectivity is achieved have not been figured out at present. Studies on the relationship between these structures and properties will broaden and deepen the applications of 0D nanomaterials.

0D nanomaterial-based biosensors play an important role in the *in vivo* bioassay. However, their application is limited due to the complexity of the *in vivo* environment. The non-specific components in the biological body affect the accuracy of detection. Furthermore, currently, most of the 0D nanomaterial-based biosensors can only be used for *in vitro* testing and laboratory experiments. As Table 1 shows, a large number of studies on disease diagnosis have been conducted, but most of them are limited to human serum, cells and animal models (mice). The lack of long-term biotoxicity evaluation and biodegradation of these materials further limit their clinical application.

**Table 1:** Summary of 0D nanomaterial-based biosensors for disease diagnosis.

**Sensor platform/label**	**Analyte**	**Experimental subject**	**Detection range**	**LOD**	**References**
afGQDs	cTnI	Blood serum	0.001–1000 ng/mL	0.192 pg/mL	Bhatnagar et al., 2016
pRF-GQDs	Solid tumors	Mice	-	-	Fan et al., 2017
N-CQDs	GLU	Blood serum	1–60 U/L	0.3 U/L	Lu et al., 2016
nano-C_60_/CdTe QDs	Thrombin (TB)	Blood serum	1 fM−10 nM	0.3 fM	Li M. et al., 2016
SnS_2_ QDs	anti-CMV pp65	Blood serum	1 fM−100 nM	0.33 fM	Lei et al., 2018
CdSe@ZnS QDs	miRNA-141	Cancer cells	100 aM−10 pM	33 aM	Li et al., 2017
V&A@Ag_2_S QDs	ONOO^−^	Mice	-	0.06 μM	Li et al., 2020
MNPs-Abs	CA-125, β2M, ApoA1	Blood serum	-	0.26 U/mL, 0.55 ng/mL, 7.7 ng/mL	Pal et al., 2015
rGO/Au	L-Cysteine	Cancer cells	-	0.51 nM	Thirumalraj et al., 2018
CPs@MnO_2_-AgNPs	GSH	Cancer cells	0.8–80 μM	0.55 μM	Wang Q. et al., 2019
UCNP@SiO_2_@Cy5-pep	Caspase-9	Mice	0.5–100 U/mL	0.068 U/mL	Liu L. et al., 2019

Despite the remarkable achievements of 0D nanomaterials in the field of biosensors, there are still some disadvantages. In Table 2, we summarize in detail the advantages and disadvantages of different 0D nanomaterials in biosensing applications. Moreover, compared with other low-dimensional nanomaterials (Li B. L. et al., 2016; Jiang et al., 2018; Chen X. et al., 2019), 0D nanomaterials have higher PL quantum yield due to the quantum confinement effect. Thus, 0D nanomaterials play a more important role in many fluorescence sensing systems. In addition, *in vivo* applications, the size advantage of 0D nanomaterials is extremely useful for cell uptake and downstream cell processing. However, soft and scalable electronic devices based on one-dimensional nanomaterials have gained rapidly increasing attention in recent years because they can perform real-time non-invasive continuous monitoring (Araki et al., 2019; Tang et al., 2019; Zhai et al., 2019; Zhou et al., 2020). Research into the manufacture of scalable biomedical sensors using 0D nanomaterials is still scarce. Furthermore, due to their ultra-small sizes, 0D nanomaterials are more difficult to regulate in the synthesis process than other low-dimensional materials.

**Table 2:** Summary of advantages and disadvantages of different 0D nanomaterial-based biosensors.

**0D nanomaterials**	**Advantages**	**Disadvantages**
GQDs	The tunable PL; high photostability against photobleaching and blinking; excellent aqueous solubility; low toxicity.	The confusing relationship between the surface chemistry and physicochemical properties; the unclear transmission mechanism.
CQDs	High chemical stability; simple and low-cost synthesis process; fast and easy functionalization; highly resistant to photobleaching; high aqueous stability.	The ambiguous of exact origin of fluorescence; the unclear role of doped ions; the unknown location of doped ions in CQDs.
Fullerenes	Broad light absorption in the UV-vis region; the ability to accommodate multiple electrons and endohedral metal atoms; high electron affinities; charge separation acceleration; good adsorption abilities to organic molecules.	High-cost synthesis; weak fluorescent emission; lack of proper chemical modification and bio-conjugation.
QDs	Low costs; facile preparation; size tunable light emission; high PL quantum yield; large Stokes shifts.	Potential toxic effects.
MNPs	Low toxicity; high saturation magnetization; stable magnetic and surface properties; rapid collection of the analyte; rapid response time for sensors; highly sensitive detection.	Easy aggregation.
AuNPs	Easy operation; simplicity of construction; easy functionalization; easy to absorb and capture of target analyte; readily enhance scattering and absorption of light; low toxicity.	Unoptimized synthesis conditions.
AgNPs	Similar physicochemical properties to AuNPs; more affordable than AuNPs; antifungal and antibacterial properties; strong plasmon resonance.	Potential toxic effects.
UCNPs	Efficient upconversion PL; resistance to photoblinking and photobleaching; minimal background autofluorescence; deep tissue penetration; low toxicity.	Low emission intensities; relatively poor upconversion efficiencies.
Pdots	Excellent biocompatibility; easy surface functionalization; high fluorescence quantum yield; high photo stability; low cytotoxicity.	Difficult to change the emission spectrum.

All in all, 0D nanomaterials have an excellent affinity with biomolecules and can promote the fixation of enzyme, antibody, proteins, nucleic acid and many other clinically relevant substances, providing the possibility for the development of a variety of biosensor platforms. 0D structures provide nanomaterials with different physical and chemical properties such as high surface-to-volume ratios, tunable optical properties, and excellent binding ability with biomolecules. As discussed in this review, the extensive application of 0D nanomaterials demonstrates their ability to increase the sensitivity and adjustability of biomolecular detection methods. There is no doubt that 0D nanomaterials will expand the field of biosensing, and enter the clinical research and application stage in the near future.

## Author Contributions

RL and MW conceived the review topic and modified the manuscript. ZW wrote the manuscript in consultation with all the other authors. TH arranged all the figures. All authors contributed to the final manuscript.

## Conflict of Interest

The authors declare that the research was conducted in the absence of any commercial or financial relationships that could be construed as a potential conflict of interest.
